# Supplementary material for: Natural Cellulose-Based Multifunctional Nanofibers for the Effective Removal of Particulate Matter and Volatile Organic Compounds
Source: Nanomaterials (Basel). 2023 May 24;13(11):1720. doi: 10.3390/nano13111720 (PMC10254128; doi:10.3390/nano13111720)
Supplement: Supplementary file 1 [file nanomaterials-13-01720-s001.zip › nanomaterials-2397472-supplementary.pdf]

Supplementary materials

Natural cellulose-based multifunctional nanofibers for the effective removal of particulate matter and volatile organic compounds

Sang Hyun Ji and Ji Sun Yun*

New Growth Materials Division, Korea Institute of Ceramic Engineering and Technology, 101, Soho-ro, Jinju, 52851, Republic of Korea

*Corresponding author.

Tel: 82-55-792-2675; Fax: 82-55-792-2651; E-mail address: susubin@kicet.re.kr (J. S. Yun)

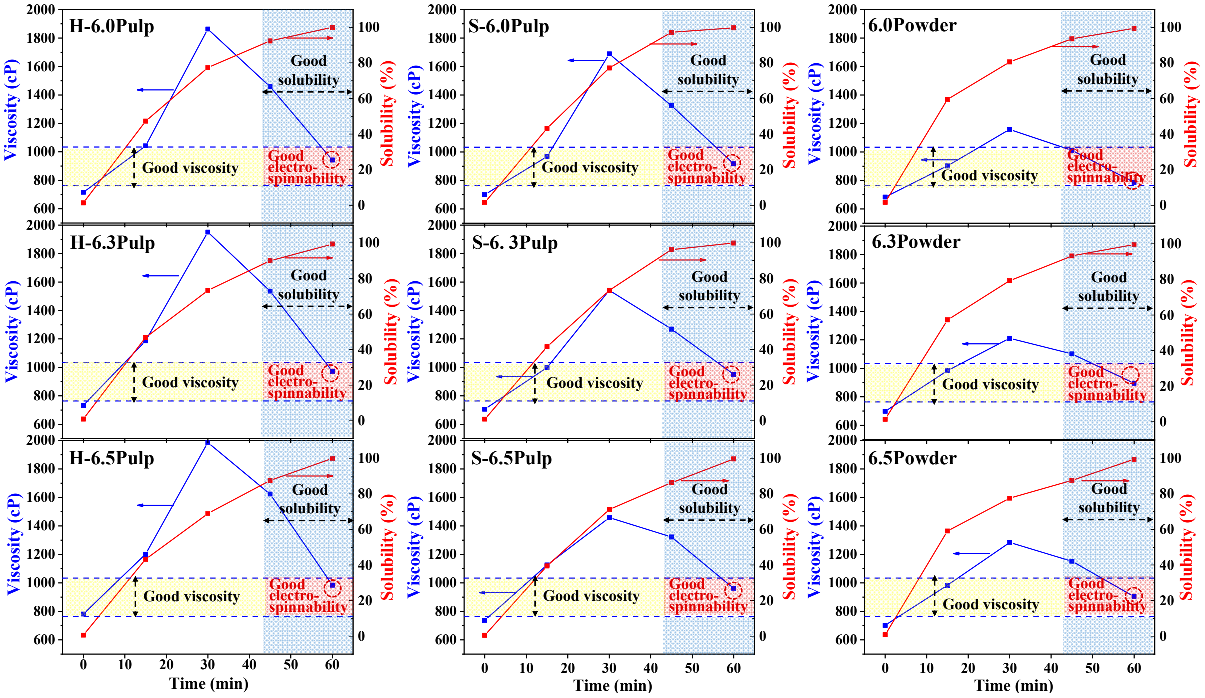

Figure S1. Viscosity and solubility characteristics of the H-pulp, S-pulp, and Powder samples with cellulose contents of 6.0, 6.3, and 6.5 wt% as a function of mixing time.

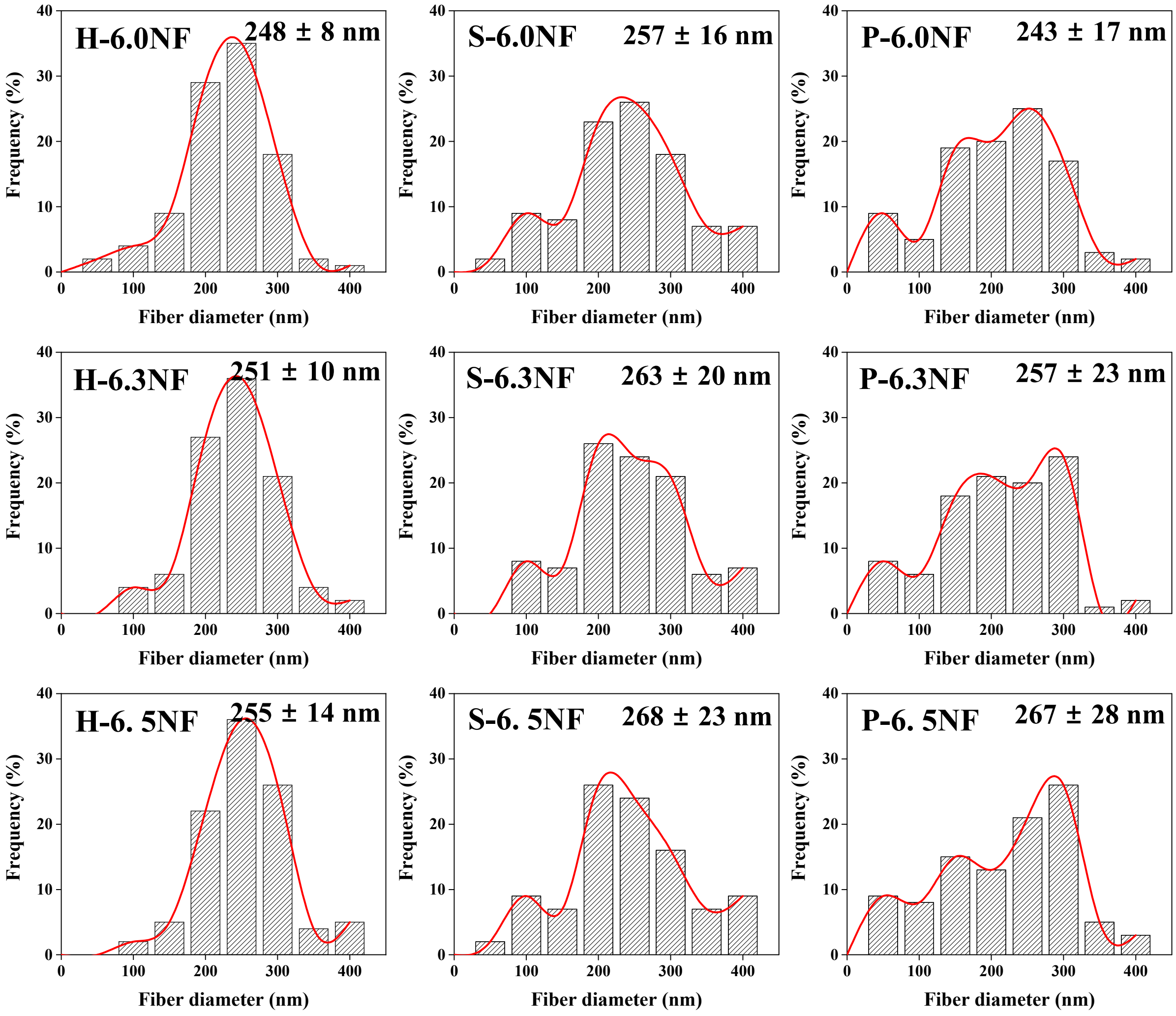

Figure S2. Characteristics of the cellulose nanofiber diameter distribution measured via the SEM images in Figure 3(a).

Table S1. Viscosity and solubility of the H-pulp, S-pulp, and Powder precursor solutions at a mixing time of 60 min.

	**Viscosity (cP)**	**Solubility (%)**
H-6.0Pulp	943	99.9
H-6.3Pulp	972	99.9
H-6.5Pulp	984	99.9
S-6.0Pulp	916	99.7
S-6.3Pulp	951	99.7
S-6.5Pulp	962	99.6
6.0Powder	786	99.5
6.3Powder	895	99.4
6.5Powder	904	99.4

Table S2. Specific surface area and pore characteristics of the H-NF, S-NF, and P-NF samples with various cellulose contents.

	**Specific surface area (m^2/g)**	**Pore size (nm)**	**Pore volume (cm^3/g)**
H-Pulp (raw)	936	1.43	0.07
H-6.0NF	1043	1.58	0.10
H-6.3NF	1053	1.61	0.11
H-6.5NF	1082	1.73	0.10
S-Pulp (raw)	848	1.41	0.07
S-6.0NF	1011	1.57	0.09
S-6.3NF	1026	1.60	0.10
S-6.5NF	1042	1.70	0.09
Powder (raw)	716	1.37	0.04
P-6.0NF	893	1.53	0.05
P-6.3NF	908	1.60	0.06
P-6.5NF	913	1.69	0.05

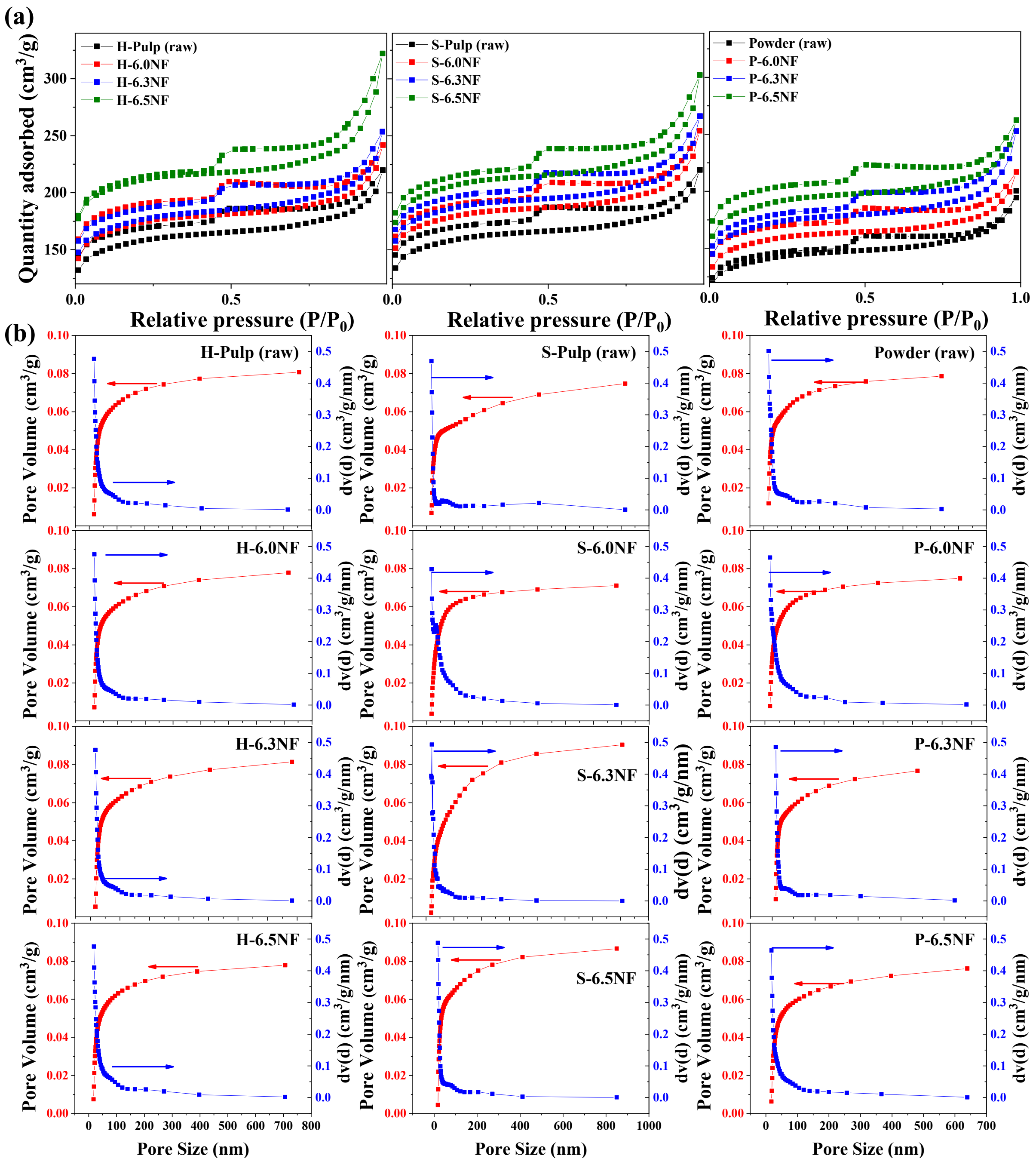

Figure S3. (a) Nitrogen sorption isotherms and (b) pore-size distribution curves of the H-NF, S-NF, and P-NF samples with various cellulose contents.
